# Pre-operative maintenance of angiotensin-converting enzyme inhibitors is not associated with acute kidney injury in cardiac surgery patients with cardio-pulmonary bypass: a propensity-matched multicentric analysis

**DOI:** 10.3389/fphar.2024.1343647

**Published:** 2024-05-01

**Authors:** Pierre Guilleminot, Stefan Andrei, Maxime Nguyen, Osama Abou-Arab, Emmanuel Besnier, Belaid Bouhemad, Pierre-Grégoire Guinot, Collaborator study group, Jean-Baptiste Anciaux

**Affiliations:** ^1^ Department of Cardiology, Dijon University Medical Centre, Dijon, France; ^2^ Department of Anaesthesiology and Critical Care Medicine, Hopital Bichat Claude Bernard, Paris, France; ^3^ Department of Anaesthesiology and Critical Care Medicine, Dijon University Medical Centre, Dijon, France; ^4^ University of Burgundy and Franche-Comté, LNC UMR1231, F-21000, Dijon, France; ^5^ Department of Anaesthesiology and Critical Care Medicine, Amiens University Medical Centre, Amiens, France; ^6^ Department of Anaesthesiology and Critical Care Medicine, Rouen University Medical Centre, Rouen, France

**Keywords:** acute kidney injury, angiotensin-converting enzyme inhibitors, cardiac surgery, cardio-pulmonary bypass, atrial fibrillation

## Abstract

**Objective:** We investigated the effects of the maintenance of angiotensin-converting enzyme inhibitors (ACE inhibitors) the day of the surgery on the incidence of postoperative acute kidney injury (AKI) and cardiac events in patients undergoing cardiac surgery.

**Methods:** We performed a multicentric observational study with propensity matching on 1,072 patients treated with ACE inhibitors. We collected their baseline demographic data, comorbidities, and operative and postoperative outcomes. AKI was defined by KDIGO (Kidney Disease: Improving Global Outcome).

**Results:** Maintenance of an ACE inhibitor was not associated with an increased risk of AKI (OR: 1.215 (CI_95%_:0.657–2.24), *p* = 0.843, 71 patients (25.1%) vs. 68 patients (24%)). Multivariate logistic regression and sensitive analysis did not demonstrate any association between ACE inhibitor maintenance and AKI, following cardiac surgery (OR: 1.03 (CI_95%_:0.81–1.3)). No statistically significant difference occurs in terms of incidence of cardiogenic shock (OR: 1.315 (CI_95%_:0.620–2.786)), stroke (OR: 3.313 (CI_95%_:0.356–27.523)), vasoplegia (OR: 0.741 (CI_95%_:0.419–1.319)), postoperative atrial fibrillation (OR: 1.710 (CI_95%_:0.936–3.122)), or mortality (OR: 2.989 (CI_95%_:0.343–26.034)). ICU and hospital length of stays did not differ (3 [2; 5] vs. 3 [2; 5] days, *p* = 0.963 and 9.5 [8; 12] vs. 10 [8; 14] days, *p* = 0.638).

**Conclusion:** Our study revealed that maintenance of ACE inhibitors on the day of the surgery was not associated with increased postoperative AKI. ACE inhibitor maintenance was also not associated with an increased rate of postoperative major cardiovascular events (arterial hypotension, cardiogenic shock, vasopressors use, stroke and death).

## Introduction

Angiotensin-converting enzyme inhibitors (ACE inhibitors) are fundamental in cardiologic practice, serving as a primary therapeutic approach for hypertension management and cardiac remodeling in patients with conditions such as coronary artery disease, heart failure, and chronic kidney disease (Pfeffer et al., 1992; Randomized placebo-controlled trial of effect of ramipril on decline in glomerular filtration rate (eGFR) and risk of terminal renal failure in proteinuric, non-diabetic nephropathy; The GISEN Group (Gruppo Italiano di Studi Epidemiologici in Nefrologia), 1997). Additionally, they play a crucial role in managing common conditions like hypertension and diabetes, becoming indispensable in cases of cardiac or renal complications (Williams et al., 2018).

Due to their involvement in the renin–angiotensin–aldosterone system, ACE inhibitors induce sustained vasodilation through a complex mechanism. However, their mechanism of action also gives rise to adverse effects, including hypotension and hyperkalemia (Randomized placebo-controlled trial of effect of ramipril on decline in eGFR and risk of terminal renal failure in proteinuric, non-diabetic nephropathy. The GISEN Group (Gruppo Italiano di Studi Epidemiologici in Nefrologia), 1997; Coca, 2020). In perioperative settings with anesthesia, fluid shifts, and blood loss, the maintenance of ACE inhibitors has been linked to an increased risk of arterial hypotension and acute kidney injury (AKI) (Kellow, 1994; Roshanov et al., 2017; Hollmann et al., 2018). Consequently, withholding ACE inhibitors for 24 h before surgery is generally recommended to prevent persistent hypotension, especially in patients treated for arterial hypertension (Sousa-Uva et al., 2018; Sahai et al., 2022).

Cardiac surgery and cardiopulmonary bypass (CPB) further complicate the perioperative scenario as patients require cardioprotective medications while being vulnerable to hemodynamic instability due to factors like heart failure, CPB, hypovolemia, and vasoplegia. These conditions can lead to tissue perfusion imbalance and organ dysfunction, notably AKI (Brown et al., 2023). The European Association of Cardiothoracic Surgery (EACTS) guidelines recommend discontinuing ACE inhibitors 24–48 h before cardiac surgery due to the risk of postoperative AKI or vasoplegic shock (Sousa-Uva et al., 2018). However, these recommendations lack definitive evidence, and some authors propose a potential protective role for ACE inhibitors (Dag et al., 2013). The debate between withholding or continuing ACE inhibitors is still ongoing (Tempe and Hasija, 2020). Moreover, it is worth noting that many of these studies were conducted in the context of older technical skills and outdated technologies, with limited hemodynamic optimization and the use of antiquated CPB devices, potentially impacting the outcomes observed (De Bono et al., 2020).

This study aims to investigate the association between the maintenance of ACE inhibitors and postoperative AKI in patients undergoing cardiac surgery with CPB. Additionally, we will explore the relationship between ACE inhibitor maintenance and mortality, as well as cardiac complications.

## Methods

### Ethics

The study was approved by the French ethical committee for human subjects (2017-A01324-49). All patients have received a written document for research purposes and gave their consent prior to participation. The study was performed in accordance with the ethics standards laid down in the 1964 Declaration of Helsinki. The manuscript was drafted according to the STROBE guidelines.

### Study design and patients

We performed a *post hoc* analysis of a multicentric study comprising four tertiary university hospitals (Dijon, Amiens, Rouen, and Strasbourg, France), including adult patients who underwent cardiac surgery with CPB between January 2017 and December 2021 (Guinot et al., 2023b).

The inclusion criteria were as follows: patient age ≥ 18 years, following angiotensin-converting enzyme inhibitor treatment, cardiac surgery with the use of CPB: coronary artery bypass graft (CABG), the surgical correction of valve disease (aortic, mitral, tricuspid, and pulmonary), combined surgery, ascending aortic disease, and others.

The non-inclusion criterion was off-pump cardiac surgery. The exclusion criteria were left ventricular assist devices and heart transplantation.

### Data collection

All data were anonymously extracted from each center’s institutional database. The following baseline patient characteristics were prospectively collected: demography (age, gender, and BMI) and comorbidities (arteriopathy, stroke, chronic obstructive pulmonary disease, and atrial fibrillation). Cardiovascular risk factors include the type of surgery (valvular surgery, CABG, combined surgery, aortic disease, and other), the presence of a critical preoperative condition, the American Society of Anesthesiology (ASA) score, and special operative status (emergency, redux, and endocarditis). Intraoperative data were also collected. EuroSCORE II (European System for Cardiac Operative Risk Evaluation) was calculated for each patient.

#### ACE inhibitor

ACE inhibitors were captopril, enalapril, lisinopril, ramipril, and perindopril. Withholding ACE was defined by discontinuation over 24 h before the surgery (Sousa-Uva et al., 2018).

#### Endpoints

The primary endpoint was the occurrence of postoperative AKI during the first week in the ICU. Secondary endpoints were death, vasopressor (norepinephrine and/or vasopressin) use, occurrence of cardiogenic shock, stroke, new-onset postoperative atrial fibrillation, and ICU and hospital stays.

### Definition of endpoints

AKI was defined as an increase in serum creatinine greater than 27 μmol/L compared with preoperative baseline or urine output less than 0.5 mL kg^-1^∙h^-1^ according to the KDIGO (Kidney disease improving global outcomes) definition (Kellum et al., 2013). Cardiogenic shock was defined as any inotropic drug requirement to obtain hemodynamic stability with left and/or right ventricular dysfunction. Postoperative atrial fibrillation was defined as a new onset of irregularly irregular heart rate in the absence of P-waves lasting at least 30 s or for the duration of the ECG recording (if < 30 s) during the postoperative period (Campbell et al., 2022). Patients were continuously monitored for the first 48–72 postoperative hours. A new episode was defined as any episode of atrial fibrillation in patients naïve to preoperative atrial fibrillation.

### Perioperative clinical management

The patients were managed based on local protocols, according to national and international guidelines. Following surgery, all patients were monitored in the intensive care unit and managed by a specialized team, which included a cardiologist trained in the care of postoperative cardiac surgery. Hemodynamic support was guided by institutional protocols to achieve mean arterial pressure > 65 mmHg, cardiac index > 2.2 L min^-1^∙m^-2^, and urine output > 0.5 mL kg^-1^∙h^-1^. They also benefited from permanent monitoring of the electrocardiogram, oxygen saturation, and central venous pressure. Biological monitoring was performed several times a day at a frequency determined by the attending physician.

### Statistical analyses

A sample size of 564 patients can demonstrate an absolute difference of 5% of AKI between the two groups (for an incidence of AKI of 25%) with a risk alpha of 0.05 and a power of 0.8. Normality was assessed visually using histograms and with the Shapiro–Wilk test. The quantitative variables were presented as mean ± standard deviation (SD) or as median [25%–75% interquartile range], as appropriate. The qualitative variables were presented as numbers (percentages).

We used a combined statistical approach to assess the association between preoperative ACE inhibitor discontinuation and postoperative AKI. First, we performed propensity score matching using a nearest-neighbor algorithm with 1:1 matching without replacement and a caliper distance of less than 0.2 of the standard deviation of the logit of the propensity score, to reduce the influence of potential confounders between the two groups. Only complete datasets were used. Seventeen covariates were selected and entered: center, age, dyslipidemia, coronary artery disease, smoking, renal function, preoperative atrial fibrillation, statin, metformin, number of procedures, valvular surgery, length of aortic clamping, glucose–insulin–potassium, hypothermia, del Nido cardioplegia, custodial cardioplegia, and perioperative blood transfusion. The choice of these variables was performed considering the already validated prognostic scores in perioperative setting (EuroSCORE II) and the clinically pertinent and non-redundant status with a higher baseline disequilibrium between the two groups. The baseline disequilibrium and the adequacy of covariate balance in the matched sample were assessed using absolute standardized mean differences (mean difference expressed in units of SD). In the matched cohort, the comparison regarding the primary endpoint between the two groups of treatment was performed using the McNemar test. The comparisons regarding the secondary endpoints between the two groups were performed using the McNemar test or the Wilcoxon signed-rank test, as appropriate. Because we tested multiple outcomes, the threshold for statistical significance was corrected by using a Bonferroni correction and set at *p* < 0.006 (0.05 divided by 8, the number of assessed outcomes). Second, we performed a sensitivity analysis using a logistical model approach and an unmatched cohort. We performed a univariable logistical model with the apparition of postoperative AKI as a dependent variable and each preoperative factor as an independent variable. To take in account the effect of the center, we include the center in the multivariate logistic regression model. Non-redundant and clinically pertinent variables with a *p*-value <0.2 in univariate analyses were introduced in a multivariable logistic regression model. The association between risk factors and AKI was evaluated by the odds ratio (OR) and its 95% confidence interval. The calibration of the final logistic model was assessed using the Hosmer–Lemeshow statistic. The validity conditions for the logistic regression were checked in order to have at least 5–10 events for each independent variable in the multivariable model. All analyses were performed using R software version 3.4.4 (R Foundation for Statistical Computing, Wien, Austria). We used the “MatchIt” package for propensity score matching.

## Results

### Baseline characteristics

**FIGURE 1:**
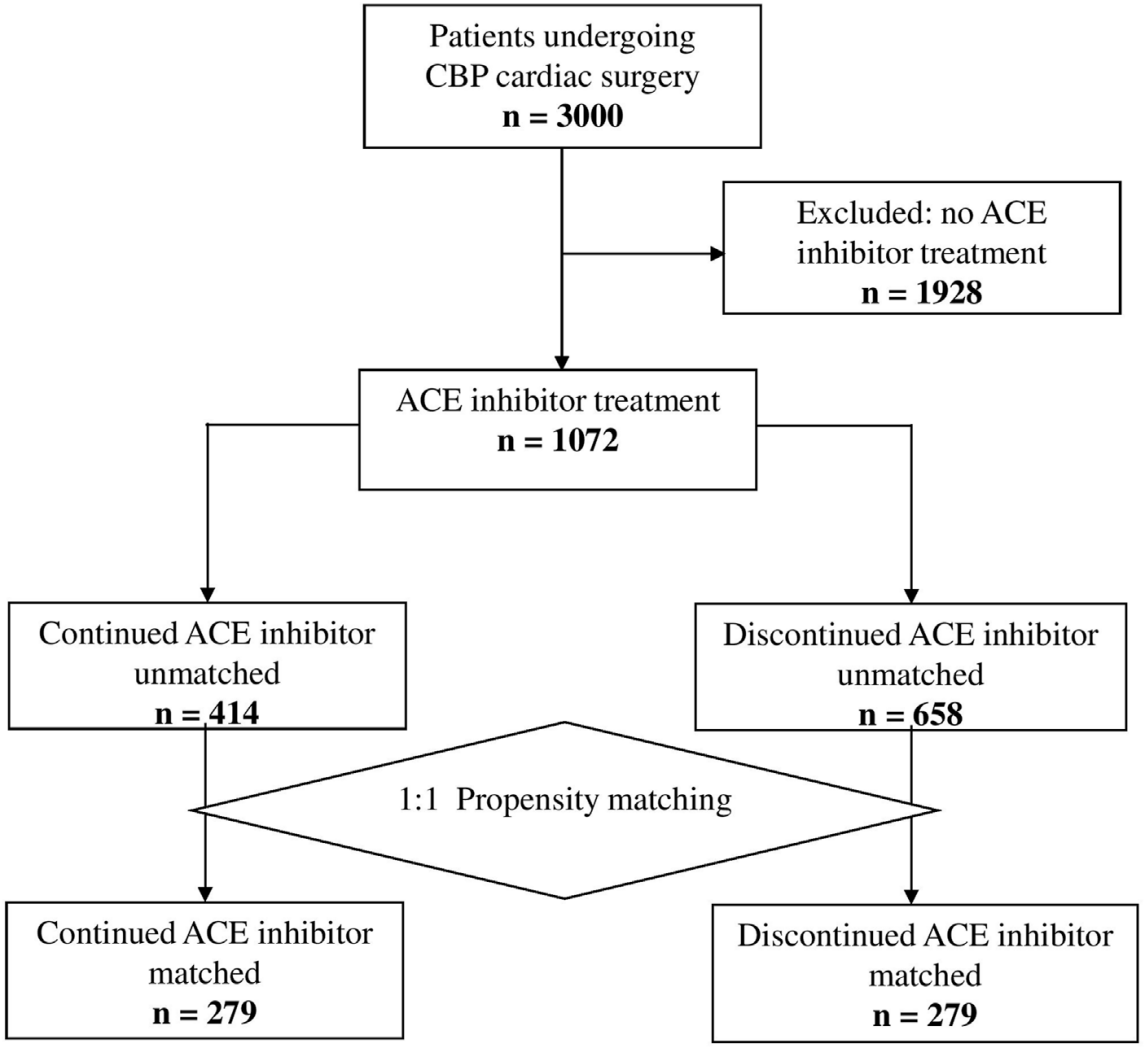
Study flowchart.

In total, 3,000 patients underwent cardiac surgery with CPB during the study period, of whom 1,072 were observing ACE inhibitor therapy. One-to-one propensity score matching has led to two matched cohorts of 283 patients (Figure 1).

The mean age was 68.6 years (SD 9.4) in the ACE inhibitor continuation group and 68.7 years (SD 9.42) in the ACE inhibitor withholding group. The mean left ventricular ejection function (LVEF) was 55% (SD 12.5 and 11.9) in both groups. The mean estimated eGFRs were 78 mL∙min^-1^∙1.73m^-2^ (SD 25.8) in the ACE inhibitor continuation group and 79 mL∙min^-1^∙1.73m^-2^ (SD 25.7) in the ACE inhibitor withholding group. The groups were considered well-balanced after matching for several variables (Table 1).

**TABLE 1:** Baseline characteristics.

	Unmatched cohort			Matched cohort		
	Maintained ACE inhibitor	Withholding ACE inhibitor	SMD	Maintained ACE inhibitor	Withholding ACE inhibitor	SMD
	*n* = 658	*n* = 414		*n* = 283	*n* = 283	
**Age (years), mean (SD)**	66.9 (10.2)	68.1 (9.5)	0.126	68.6 (9.9)	68.7 (9.4)	0.010
**BMI kg.m** ^**-2**^ **, mean (SD)**	27.79 (5.12)	29.08 (21.62)	0.082	27.60 (5.00)	28.02 (5.09)	0.083
**Gender (male), n (%)**	501 (76.1)	304 (73.4)	0.062	203 (71.7)	205 (72.4)	0.016
**EuroSCORE 2, mean (SD)**	4.27 (7.97)	4.23 (7.45)	0.004	4.89 (8.52)	4.92 (8.48)	0.003
**High blood pressure, n (%)**	513 (78.0)	314 (75.8)	0.050	218 (77.0)	216 (76.3)	0.017
Diabetes, n (%)						
- **Type 1**	49 (7.4)	25 (6.0)	0.056	24 (8.5)	23 (8.1)	0.013
- **Type 2**	164 (24.9)	121 (29.2)	0.097	78 (27.6)	86 (30.4)	0.062
**Dyslipidemia, n (%)**	345 (52.4)	193 (46.6)	0.116	128 (45.2)	136 (48.1)	0.057
Arteriopathy, n (%)						
- **Peripheral artery disease**	84 (12.8)	55 (13.3)	0.015	36 (12.7)	40 (14.1)	0.041
- **Abdominal aortic aneurysm**	22 (3.3)	7 (1.7)	0.106	9 (3.2)	6 (2.1)	0.066
- **Carotid stenosis**	45 (6.8)	24 (5.8)	0.043	26 (9.2)	20 (7.1)	0.078
**Coronary artery disease, n (%)**	362 (55.0)	252 (60.9)	0.119	170 (60.1)	174 (61.5)	0.029
**Chronic renal failure, n (%)**	106 (16.1)	82 (19.8)	0.096	50 (17.7)	57 (20.1)	0.063
**Smoking, n (%)**	217 (33.0)	94 (22.7)	0.231	91 (32.2)	93 (32.9)	0.015
**Chronic obstructive pulmonary disease, n (%)**	46 (7.0)	19 (4.6)	0.141	20 (7.1)	18 (6.4)	0.028
**Stroke, n (%)**	66 (10.0)	37 (8.9)	0.037	41 (14.5)	28 (9.9)	0.141
**Atrial fibrillation, n (%)**	109 (16.6)	86 (20.8)	0.108	75 (26.5)	61 (21.6)	0.116
**Critical state, n (%)**	34 (5.2)	27 (6.5)	0.058	17 (6.0)	24 (8.5)	0.096
**Intra-aortic balloon pump, n (%)**	6 (0.9)	3 (0.7)	0.021	1 (0.4)	3 (1.1)	0.084
**Right ventricular dysfunction, n (%)**	31 (4.7)	14 (3.4)	0.068	17 (6.0)	14 (4.9)	0.047
**LVEF (%), (mean (SD))**	55.2 (11.7)	55.7 (11.6)	0.044	54.1 (12.5)	54.5 (11.9)	0.034
PHT, n (%)						
- **Moderate (30–50** ** ** **mmHg)**	122 (18.5)	73 (17.6)	0.024	64 (22.6)	54 (19.1)	0.087
- **Severe (>50** ** ** **mmHg**)	74 (11.2)	45 (10.9)	0.012	33 (11.7)	31 (11.0)	0.022
**eGFR (ml∙min** ^**-1**^ **∙1.73m** ^**-2**^ **), mean (SD)**	80.79 (26.73)	77.19 (26.53)	0.135	78.23 (25.79)	79.02 (25.73)	0.030
*Medications*						
**Beta-blocker, n (%)**	465 (70.7)	285 (68.8)	0.040	213 (75.3)	194 (68.6)	0.150
**Calcium channel blocker, n (%)**	155 (23.6)	99 (23.9)	0.008	67 (23.7)	70 (24.7)	0.025
**Amiodaron, n (%)**	30 (4.6)	32 (7.7)	0.132	23 (8.1)	18 (6.4)	0.068
**Aspirin, n (%)**	395 (60.0)	271 (65.5)	0.112	175 (61.8)	188 (66.4)	0.096
**Aldosterone antagonist, n (%)**	36 (5.5)	19 (4.6)	0.040	20 (7.1)	16 (5.7)	0.058
**P2Y12 inhibitors, n (%)**	70 (10.6)	54 (13.0)	0.074	31 (11.0)	34 (12.0)	0.033
**Statin, n (%)**	423 (64.3)	242 (58.5)	0.120	164 (58.0)	171 (60.4)	0.050
**Loop diuretic, n (%)**	168 (25.5)	116 (28.0)	0.056	76 (26.9)	78 (27.6)	0.016
**Metformin, n (%)**	63 (9.6)	58 (14.0)	0.138	40 (14.1)	40 (14.1)	<0.001
**Insulin, n (%)**	48 (7.3)	31 (7.5)	0.007	25 (8.8)	23 (8.1)	0.025
*Operative*						
**Emergency, n (%)**	96 (14.6)	69 (16.7)	0.057	44 (15.5)	57 (20.1)	0.120
**Redo surgery, n (%)**	31 (4.7)	18 (4.3)	0.017	16 (5.7)	12 (4.2)	0.065
**Endocarditis, n (%)**	19 (2.9)	9 (2.2)	0.045	9 (3.2)	6 (2.1)	0.066
**Number of procedures (mean (SD))**	1.39 (0.63)	1.30 (0.53)	0.149	1.34 (0.59)	1.32 (0.55)	0.025
**Valvular surgery, n (%)**	316 (48.0)	211 (51.0)	0.059	131 (46.3)	131 (46.3)	<0.001
**Only valvular surgery, n (%)**	131 (19.9)	113 (27.3)	0.175	58 (20.5)	64 (22.6)	0.052
**Mitral, n (%)**	96 (14.6)	55 (13.3)	0.038	43 (15.2)	31 (11.0)	0.126
**Aortic, n (%)**	239 (36.3)	162 (39.1)	0.058	95 (33.6)	104 (36.7)	0.067
**Tricuspid, n (%)**	31 (4.7)	17 (4.1)	0.029	15 (5.3)	9 (3.2)	0.105
**Only CABG, n (%)**	300 (45.6)	184 (44.4)	0.023	134 (47.3)	134 (47.3)	<0.001
**Combined surgery, n (%)**	204 (31.0)	111 (26.8)	0.093	82 (29.0)	80 (28.3)	0.016
**Ascending aorta, n (%)**	66 (10.0)	32 (7.7)	0.081	21 (7.4)	24 (8.5)	0.039
**Other type, n (%)**	62 (9.4)	27 (6.5)	0.107	33 (11.7)	24 (8.5)	0.106
**Length of aortic clamping (min), (mean SD)**	84.8 (38.3)	79.6 (35.3)	0.142	80.5 (36.6)	80.3 (34.6)	0.003
**Hypothermia, n (%)**	420 (63.8)	192 (47.1)	0.342	147 (51.9)	155 (54.8)	0.057
**Glucose, insulin, potassium, n (%)**	199 (30.2%)	86 (20.8%)	0.365	90 (31.8)	85 (30.0)	0.038
**Blood cardioplegia, n (%)**	225 (34.5)	156 (39.6)	0.107	133 (47.0)	131 (46.3)	0.014
**Custodiol cardioplegia, n (%)**	80 (12.3)	30 (7.7)	0.153	30 (10.6)	24 (8.5)	0.072
**Del Nido cardioplegia, n (%)**	325 (49.8)	114 (29.2)	0.431	104 (36.7)	114 (40.3)	0.073
**Type of anesthesia, n (%)**						0.122
- **OFA**	312 (40.7)	201 (48.6)		125 (44.2)	109 (38.5)	
- **Opioid use**	390 (59.3)	213 (50.5)	0.158	158 (55.8)	175 (61.5)	
**Blood transfusion, n (%)**	137 (20.8)	113 (27.3)	0.152	63 (22.6)	63 (22.6)	<0.001

BMI, body mass index; CABG, coronary artery bypass graft; LVEF, left ventricular ejection fraction; OFA, opioid-free anesthesia; PHT, pulmonary hypertension.

### Postoperative outcomes

**FIGURE 2:**
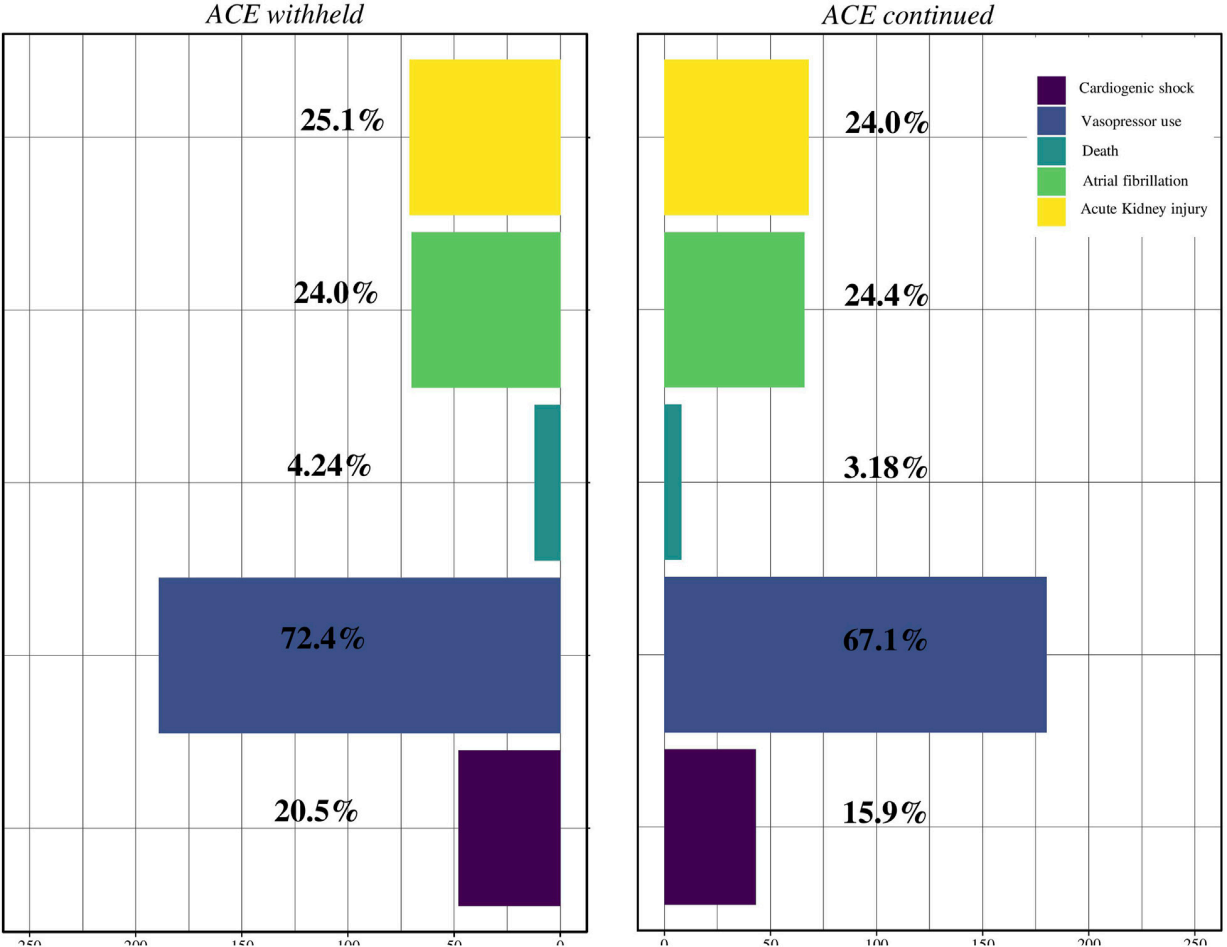
Incidence of postoperative acute kidney injury, death, cardiogenic shock, stroke, and vasopressor use according to angiotensin-converting enzyme (ACE) inhibitor maintenance or withholding.

Seventy-one (25.1%) patients developed postoperative AKI in the withholding ACE inhibitor group and sixty-eight (24%) in the ACE inhibitor maintenance group, which was not a statistically significant difference (*p* = 0.843). The postoperative outcomes are presented in Tables 2, 3 and Figure 2, according to ACE inhibitor group management.

**TABLE 2:** Postoperative outcomes in the matched cohort stratified by angiotensin-converting enzyme (ACE) inhibitor exposure.

	Maintained ACE inhibitor	Withholding ACE inhibitor	*p*-value
	*n* = 283	*n* = 283	
*Primary endpoint*			
AKI, n (%)	68 (24.0%)	71 (25.1%)	0.843
Dialysis, n (%)	12 (4.24%)	9 (3.18%)	0.662
*Secondary endpoints*			
Vasopressor use, n (%)	190 (67.1%)	205 (72.4%)	0.214
Cardiogenic shock, n (%)	45 (15.9%)	58 (20.5%)	0.184
Postoperative AF, n (%)	69 (24.4%)	68 (24.0%)	1
Stroke, n (%)	13 (4.59%)	8 (2.83%)	0.358
Death, n (%)	9 (3.18%)	12 (4.24%)	0.646
ICU Length of stay (days), [IQR]	3 [2; 5]	3 [2; 5]	0.963
Hospital Length of stay (days), [IQR]	10 [8; 14]	9.5 [8; 12]	0.638

**TABLE 3:** Odds ratio for postoperative outcomes stratified by angiotensin-converting enzyme (ACE) inhibitor exposure.

	Unadjusted OR (95%CI)	Propensity score matching (PSM)-adjusted OR (95%CI)
AKI, n (%)	0.92 [0.83; 1.43] *p* = 0.191	1.22 [0.66–2.24] *p* = 0.843
Dialysis, n (%)	1.24 [0.64; 2.37] *p* = 0.518	0.49 [0.11–2.18] *p* = 0.662
Vasopressor use, n (%)	0.56 [0.43; 0.73] *p* = 0.001	0.74 [0.42–1.32] *p* = 0.214
Cardiogenic shock, n (%)	1.03 [0.74; 1.42] *p* = 0.881	1.32 [0.62–2.79] *p* = 0.184
Postoperative Atrial fibrillation (AF), n (%)	0.71 [0.53; 0.95] *p* = 0.022	1.70 [0.94–3.12] *p* = 1
Stroke, n (%)	1.51 [0.73; 3.12] *p* = 0.266	3.31 [0.36–27.52] *p* = 0.358
Death, n (%)	0.66 [0.30; 1.36] *p* = 0.269	2.99 [0.33–26.03] *p* = 0.646

We did not observe a statistically significant difference in terms of mortality (3.18% vs 4.24%, *p* = 0.646). In addition, there was no statistically significant difference in postoperative atrial fibrillation (24.4% vs 24.0%, *p* = 1) and postoperative cardiogenic shock (15.9% vs 20.5%, *p* = 0.184), despite the group. The incidence of severe hypotension requiring vasopressor use was similar between the two groups (72,4% vs. 67,1%, *p* = 0.214). Finally, the ICU and hospital length of stays did not differ between the two groups (3 [2; 5] vs. 3 [2; 5] days, *p* = 0.963 and 9.5 [8; 12] vs. 10 [8; 14] days, *p* = 0.638).

### Sensitivity analysis and postoperative AKI predictors

**FIGURE 3:**
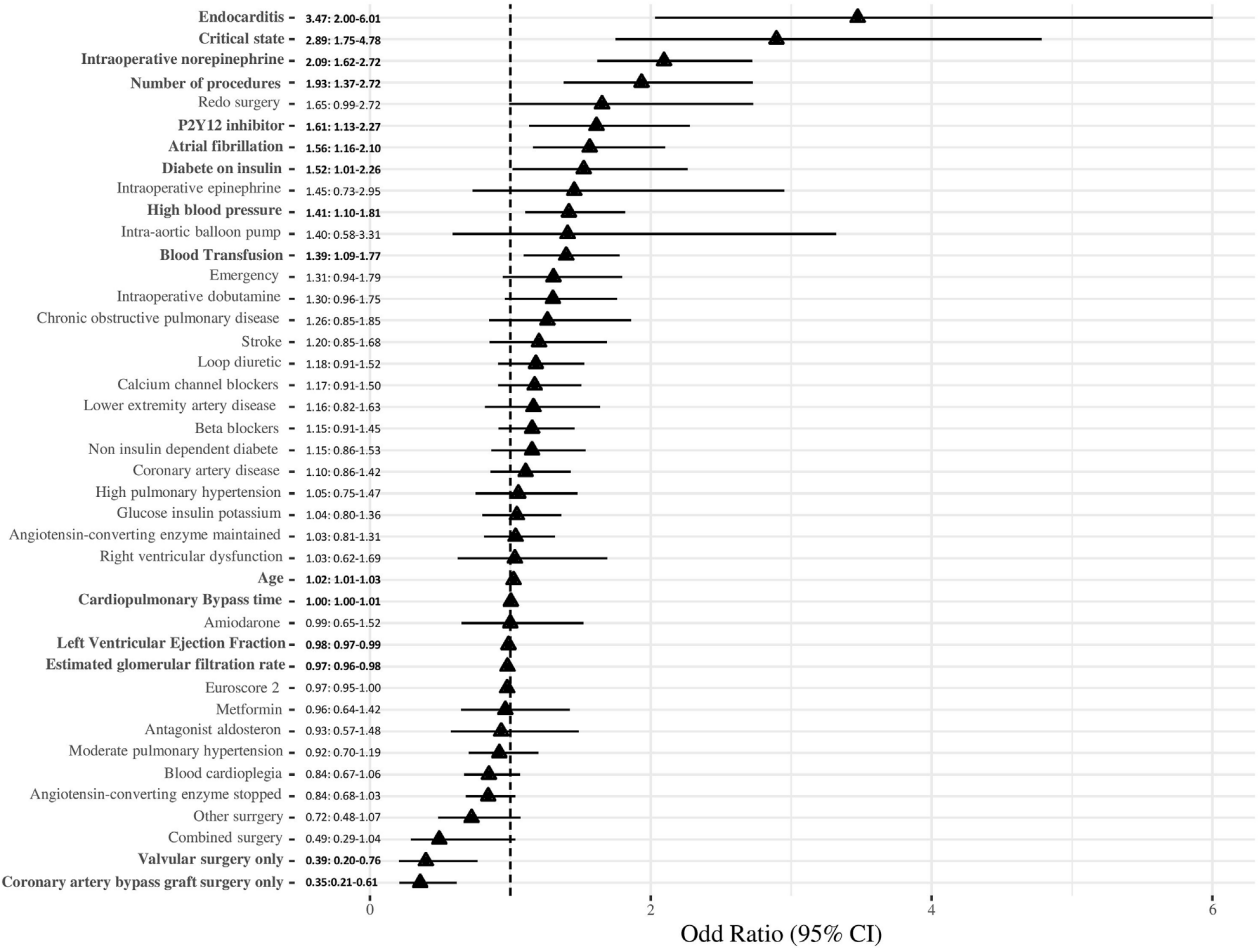
Forrest plot of factors associated with angiotensin-converting enzyme (AKI). The odds ratio and their 95% confidence interval are presented in the figure. Variables in bold have a significant association (*p* < 0.05).

The continuation of the ACE inhibitor was not associated with postoperative AKI in univariable logistic regression (OR: 1.2 (0.9–1.5), *p* = 0.246). The logistic regression results regarding the factors associated with postoperative AKI are presented in Figure 3. These factors comprise age, high blood pressure, atrial fibrillation, diabetes on insulin, critical state, LVEF, estimated eGFR, P2Y12 inhibitor use, endocarditis, number of procedures, only valvular surgery, only CABG surgery, length of CPB time, norepinephrine use, and intraoperative blood transfusion.

## Discussion

Our study sheds light on the preoperative management of ACE inhibitors in cardiac surgery patients. Notably, our research revealed that continuing ACE inhibitors neither exhibited a significant association with postoperative AKI or postoperative cardiac events, such as vasopressor use, postoperative atrial fibrillation, or stroke nor did they impact the length of hospital stays. These findings challenge prior assumptions and underline the need for a nuanced understanding of ACE inhibitor use in contemporary cardiac surgical practices.

To interpret our results effectively, it is crucial to consider the broader literature landscape, often comprising retrospective studies focusing on the mere use or non-use of ACE inhibitors rather than their maintenance or discontinuation. Historical studies indicated increased postoperative AKI and mortality (Miceli et al., 2009; Bandeali et al., 2012; Yacoub et al., 2013), prompting guidelines from bodies like the European Association for Cardio-Thoracic Surgery (EACTS), with the support of the meta-analysis of Zhang and Ma (Zhang and Ma, 2015), to recommend withholding ACE inhibitors before cardiac surgery in 2017 (Sousa-Uva et al., 2018). However, subsequent research, including small, randomized trials, failed to establish a definitive correlation between ACE inhibitor maintenance and major cardiac events (van Diepen et al., 2018). Interestingly, studies showcasing a decrease in postoperative AKI in CABG patients continuing ACE inhibitors contrasted with our findings (Benedetto et al., 2008; Drenger et al., 2012; Dag et al., 2013).

Our findings do not favor any specific strategy regarding preoperative ACE inhibitor management; however, they did not demonstrate an increased risk of AKI with the maintenance of ACE inhibitors. Several factors might explain our results. Notably, our study focused on modern cardiac care, where physicians employ proactive hemodynamic strategies and optimize cardiovascular states (Patel et al., 2020), potentially mitigating perioperative risks associated with ACE inhibitor maintenance. These strategies are part of enhanced recovery after surgery (ERAS) programs and are widely recommended (Mertes et al., 2022). These results, therefore, corroborate a previous pilot randomized controlled trial conducted by van Diepen et al. (2018). van Diepen et al. observed similar rates of events and vasopressor usage (approximately 70%) between patients with the continuation or withholding of ACE inhibitors. Taken together, these results suggest that medical strategies aimed at optimizing blood pressure, cardiac output, and fluid balance may play a more significant role in the pathogenesis of AKI during cardiac surgery than the use and/or maintenance of ACE inhibitors. Although our findings do not endorse a specific preoperative ACE inhibitor management strategy, they emphasize the importance of individualized patient care, particularly in high-risk cases (e.g., chronic renal impairment, atrial fibrillation, infective endocarditis, and severe heart failure), as recommended by international guidelines. Moreover, our results confirm that these factors remain associated with AKI (Peng et al., 2022).

Although our study challenges the routine withholding of ACE inhibitors, interpreting these findings requires caution. The rapidly evolving landscape of cardiac surgery, encompassing advancements in monitoring, surgical techniques, cardioplegia, and anesthesia, may influence postoperative outcomes (Guinot et al., 2019; 2023a). Future studies should consider the impact of postoperative reintroduction timing of ACE inhibitors, possibly in conjunction with other medications like beta-blockers, to reconcile the variability observed across the published literature. Interestingly, the perioperative management strategy of ACE inhibitors was not associated with other cardiovascular events such as atrial fibrillation or vasoplegia. Older studies suggested a contradictory association between these major cardiac events (Miceli et al., 2009; Bandeali et al., 2012). However, we did not consider the potential mediation of the antiarrhythmic effect by ACE insertion/deletion polymorphism.

### Limitations

Because of its retrospective nature, we do not know the dosage and the type of molecule used. The use of the KDIGO definition, which gives a central place to serum creatinine in the definition of AKI, is not necessarily a reflection of structural renal damage (Coca et al., 2014). Cola et al. had demonstrated that despite an increase in postoperative creatinine levels, urinary biomarkers of tubular damage did not increase in patients who continued ACE inhibitors. It seems legitimate to ask whether the follow-up period is sufficient. Finally, it might be interesting to study the rate of long-term ACE inhibitor treatment in cases of temporary withdrawal for cardiac surgery. Indeed, analysis of the data from Antoniak’s study on American veterans shows a lower rate of long-term ACE inhibitor treatment in cases of temporary withdrawal, which could have a negative impact on the prognosis of patients (Antoniak et al., 2021).

## Conclusion

Our study demonstrated that ACE inhibitor maintenance on the day of cardiac surgery with CPB was not associated with increased postoperative AKI. The continuation of ACE inhibitors was also not associated with an increase in the rate of major cardiac events. These results led to a reconsideration of the current recommendations on the perioperative management of ACE inhibitors, which need to be confirmed by further randomized controlled trials.

## Data Availability

The raw data supporting the conclusion of this article will be made available by the authors without undue reservation.

## Group members of Collaborator study group

Jean‐Baptiste Anciaux, Annouck Appriou, Vivien Berthoud, Tiberiu Constandache, Valerian Duclos, Sandrine Grosjean, Audrey Martin, Mohamed Radhouani, Bastien Durand (Department of Anaesthesiology and Critical Care Medicine, Dijon University Medical Centre, Dijon, France); Christophe Beyls, Hervé Dupont (Department of Anaesthesiology and Critical Care Medicine, Amiens University Medical Centre, Amiens, France); Chloe Bernard, Olivier Bouchot, Saed Jazayeri, Marie‐Catherine Morgant (Department of Cardiac Surgery, Dijon University Medical Centre, Dijon, France); Michel Kindo (Department of Cardiovascular Surgery, University Hospitals of Strasbourg, Strasbourg, France); Paul Michel Mertes (Department of Anaesthesiology and Critical Care Medicine, Strasbourg University Medical Centre, Strasbourg, France).

## Abbreviations

ACE, angiotensin-converting enzyme; AKI, acute kidney injury; CABG, coronary artery bypass graft; CPB, cardiopulmonary bypass; eGFR, glomerular filtration rate; LVEF, left ventricular ejection function.

## References

[B30] Antoniak D. T. Walters R. W. Alla V.M. (2021). Impact of Renin-Angiotensin System Blockers on Mortality in Veterans Undergoing Cardiac Surgery. J. Am. Heart Assoc. 10 (10), e019731. 10.1161/JAHA.120.019731 33969701 PMC8200704

[B1] Bandeali S. J. Kayani W. T. Lee V.-V. Pan W. Elayda M. A. A. Nambi V. (2012). Outcomes of preoperative angiotensin-converting enzyme inhibitor therapy in patients undergoing isolated coronary artery bypass grafting. Am. J. Cardiol. 110, 919–923. 10.1016/j.amjcard.2012.05.021 22727178

[B28] Benedetto U Sciarretta S Roscitano A Fiorani B Refice S Angeloni E Sinatra R. (2008). Preoperative Angiotensin-converting enzyme inhibitors and acute kidney injury after coronary artery bypass grafting. Ann Thorac Surg. 86(4):1160–1165. 10.1016/j.athoracsur.2008.06.018 18805152

[B2] Brown J. K. Shaw A. D. Mythen M. G. Guzzi L. Reddy V. S. Crisafi C. (2023). Adult cardiac surgery-associated acute kidney injury: joint consensus report. J. Cardiothorac. Vasc. Anesth. 37, 1579–1590. 10.1053/j.jvca.2023.05.032 37355415

[B3] Campbell N. G. Wollborn J. Fields K. G. Lip G. Y. H. Ruetzler K. Muehlschlegel J. D. (2022). Inconsistent methodology as a barrier to meaningful research outputs from studies of atrial fibrillation after cardiac surgery. J. Cardiothorac. Vasc. Anesth. 36, 739–745. 10.1053/j.jvca.2021.10.009 34763979 PMC9901359

[B4] Coca S. G. (2020). Angiotensin-converting enzyme inhibitors and angiotensin receptor blockers after acute kidney injury: friend, foe, or acquaintance? Am. J. Nephrol. 51, 263–265. 10.1159/000505896 32074594

[B5] Coca S. G. Garg A. X. Thiessen-Philbrook H. Koyner J. L. Patel U. D. Krumholz H. M. (2014). Urinary biomarkers of AKI and mortality 3 years after cardiac surgery. J. Am. Soc. Nephrol. 25, 1063–1071. 10.1681/ASN.2013070742 24357673 PMC4005309

[B6] Dag O. Kaygin M. A. Aydin A. Limandal H. K. Arslan Ü. Kiymaz A. (2013). Is administration of preoperative angiotensin-converting enzyme inhibitors important for renal protection after cardiac surgery? Ren. Fail 35, 754–760. 10.3109/0886022X.2013.777891 23521631

[B7] De Bono J. A. Conte S. M. Newcomb A. E. (2020). Effects of preoperative angiotensin-converting enzyme inhibitor therapy on postoperative renal function in cardiac surgery. Heart Lung Circ. 29, 1656–1667. 10.1016/j.hlc.2020.06.003 32732124

[B29] Drenger B Fontes ML Miao Y Mathew JP Gozal Y Aronson S. (2012). Patterns of use of perioperative angiotensin-converting enzyme inhibitors in coronary artery bypass graft surgery with cardiopulmonary bypass: effects on in-hospital morbidity and mortality. Circulation. 126(3):261–269. 10.1161/CIRCULATIONAHA.111.059527 22715473

[B8] Guinot P.-G. Andrei S. Durand B. Martin A. Duclos V. Spitz A. (2023a). Balanced nonopioid general anesthesia with lidocaine is associated with lower postoperative complications compared with balanced opioid general anesthesia with sufentanil for cardiac surgery with cardiopulmonary bypass: a propensity matched cohort study. Anesth. Analg. 136, 965–974. 10.1213/ANE.0000000000006383 36763521

[B9] Guinot P.-G. Durand B. Besnier E. Mertes P.-M. Bernard C. Nguyen M. (2023b). Epidemiology, risk factors and outcomes of norepinephrine use in cardiac surgery with cardiopulmonary bypass: a multicentric prospective study. Anaesth. Crit. Care Pain Med. 42, 101200. 10.1016/j.accpm.2023.101200 36758855

[B10] Guinot P.-G. Spitz A. Berthoud V. Ellouze O. Missaoui A. Constandache T. (2019). Effect of opioid-free anaesthesia on post-operative period in cardiac surgery: a retrospective matched case-control study. BMC Anesthesiol. 19, 136. 10.1186/s12871-019-0802-y 31366330 PMC6668113

[B11] Hollmann C. Fernandes N. L. Biccard B. M. (2018). A systematic review of outcomes associated with withholding or continuing angiotensin-converting enzyme inhibitors and angiotensin receptor blockers before noncardiac surgery. Anesth. Analg. 127, 678–687. 10.1213/ANE.0000000000002837 29381513

[B12] Kellow N. H. (1994). The renin-angiotensin system and angiotensin converting enzyme (ACE) inhibitors. Anaesthesia 49, 613–622. 10.1111/j.1365-2044.1994.tb14234.x 8042731

[B13] Kellum J. A. Lameire N. KDIGO AKI Guideline Work Group (2013). Diagnosis, evaluation, and management of acute kidney injury: a KDIGO summary (Part 1). Crit. Care 17, 204. 10.1186/cc11454 23394211 PMC4057151

[B14] Mertes P.-M. Kindo M. Amour J. Baufreton C. Camilleri L. Caus T. (2022). Guidelines on enhanced recovery after cardiac surgery under cardiopulmonary bypass or off-pump. Anaesth. Crit. Care Pain Med. 41, 101059. 10.1016/j.accpm.2022.101059 35504126

[B15] Miceli A. Capoun R. Fino C. Narayan P. Bryan A. J. Angelini G. D. (2009). Effects of angiotensin-converting enzyme inhibitor therapy on clinical outcome in patients undergoing coronary artery bypass grafting. J. Am. Coll. Cardiol. 54, 1778–1784. 10.1016/j.jacc.2009.07.008 19682819

[B16] Patel H. Parikh N. Shah R. Patel R. Thosani R. Shah P. (2020). Effect of goal-directed hemodynamic therapy in postcardiac surgery patients. Indian J. Crit. Care Med. 24, 321–326. 10.5005/jp-journals-10071-23427 32728322 PMC7358857

[B17] Peng K. McIlroy D. R. Bollen B. A. Billings F. T. Zarbock A. Popescu W. M. (2022). Society of cardiovascular anesthesiologists clinical practice update for management of acute kidney injury associated with cardiac surgery. Anesth. Analg. 135, 744–756. 10.1213/ANE.0000000000006068 35544772

[B18] Pfeffer M. A. Braunwald E. Moyé L. A. Basta L. Brown E. J. Cuddy T. E. (1992). Effect of captopril on mortality and morbidity in patients with left ventricular dysfunction after myocardial infarction. Results of the survival and ventricular enlargement trial. The SAVE Investigators. N. Engl. J. Med. 327, 669–677. 10.1056/NEJM199209033271001 1386652

[B19] Roshanov P. S. Rochwerg B. Patel A. Salehian O. Duceppe E. Belley-Côté E. P. (2017). Withholding versus continuing angiotensin-converting enzyme inhibitors or angiotensin II receptor blockers before noncardiac surgery: an analysis of the vascular events in noncardiac surgery patIents cOhort evaluatioN prospective cohort. Anesthesiology 126, 16–27. 10.1097/ALN.0000000000001404 27775997

[B20] Sahai S. K. Balonov K. Bentov N. Bierle D. M. M. Browning L. M. Cummings K. C. (2022). Preoperative management of cardiovascular medications: a society for perioperative assessment and quality improvement (spaqi) consensus statement. Mayo Clin. Proc. 97, 1734–1751. 10.1016/j.mayocp.2022.03.039 36058586

[B21] Sousa-Uva M. Head S. J. Milojevic M. Collet J.-P. Landoni G. Castella M. (2018). 2017 EACTS Guidelines on perioperative medication in adult cardiac surgery. Eur. J. Cardiothorac. Surg. 53, 5–33. 10.1093/ejcts/ezx314 29029110

[B22] Tempe D. K. Hasija S. (2020). Con: does preoperative discontinuation of angiotensin-converting enzyme inhibitors/angiotensin II receptor blockers reduce postoperative acute kidney injury? J. Cardiothorac. Vasc. Anesth. 34, 2836–2838. 10.1053/j.jvca.2020.03.025 32444301

[B23] The GISEN Group (Gruppo Italiano di Studi Epidemiologici in Nefrologia) (1997). Randomised placebo-controlled trial of effect of ramipril on decline in glomerular filtration rate and risk of terminal renal failure in proteinuric, non-diabetic nephropathy. The GISEN Group (Gruppo Italiano di Studi Epidemiologici in Nefrologia). Lancet 349, 1857–1863. 9217756

[B24] van Diepen S. Norris C. M. Zheng Y. Nagendran J. Graham M. M. Gaete Ortega D. (2018). Comparison of angiotensin-converting enzyme inhibitor and angiotensin receptor blocker management strategies before cardiac surgery: a pilot randomized controlled registry trial. J. Am. Heart Assoc. 7, e009917. 10.1161/JAHA.118.009917 30371293 PMC6474971

[B25] Williams B. Mancia G. Spiering W. Agabiti Rosei E. Azizi M. Burnier M. (2018). 2018 ESC/ESH Guidelines for the management of arterial hypertension. Eur. Heart J. 39, 3021–3104. 10.1093/eurheartj/ehy339 30165516

[B26] Yacoub R. Patel N. Lohr J. W. Rajagopalan S. Nader N. Arora P. (2013). Acute kidney injury and death associated with renin angiotensin system blockade in cardiothoracic surgery: a meta-analysis of observational studies. Am. J. Kidney Dis. 62, 1077–1086. 10.1053/j.ajkd.2013.04.018 23791246

[B27] Zhang Y. Ma L. (2015). Effect of preoperative angiotensin-converting enzyme inhibitor on the outcome of coronary artery bypass graft surgery. Eur. J. Cardiothorac. Surg. 47, 788–795. 10.1093/ejcts/ezu298 25079771

